# Efficient Molecular Crystal Structure Prediction and Stability Assessment with AIMNet2 Neural Network Potentials

**DOI:** 10.1021/acs.cgd.5c01001

**Published:** 2025-10-14

**Authors:** Kamal Singh Nayal, Dana O’Connor, Roman Zubatyuk, Dylan M. Anstine, Yi Yang, Rithwik Tom, Wenda Deng, Kehan Tang, Noa Marom, Olexandr Isayev

**Affiliations:** † Department of Chemistry, 6612 Carnegie Mellon University, 5000 Forbes Avenue, Pittsburgh, Pennsylvania 15213, United States; ‡ Department of Materials Science and Engineering, Carnegie Mellon University, 5000 Forbes Avenue, Pittsburgh, Pennsylvania 15213, United States; § Department of Physics, Carnegie Mellon University, 5000 Forbes Avenue, Pittsburgh, Pennsylvania 15213, United States

## Abstract

Identifying thermodynamically stable crystal structures remains a key challenge in materials chemistry. Computational crystal structure prediction (CSP) workflows typically rank candidate structures by lattice energy to assess relative stability. Approaches using self-consistent first-principles calculations become prohibitively expensive, especially when millions of energy evaluations are required for complex molecular systems with many atoms per unit cell. Here, we provide a detailed analysis of our methodology and results from the seventh blind test of crystal structure prediction organized by the Cambridge Crystallographic Data Centre (CCDC). We present an approach that significantly accelerates CSP by training target-specific machine-learned interatomic potentials (MLIPs). AIMNet2 MLIPs are trained on density functional theory (DFT) calculations of molecular clusters, herein referred to as *n*-mers. We demonstrate that potentials trained on gas phase dispersion-corrected DFT reference data of *n*-mers successfully extend to crystalline environments, accurately characterizing the CSP landscape and correctly ranking structures by relative stability. Our methodology effectively captures the underlying physics of thermodynamic crystal stability using only molecular cluster data, avoiding the need for expensive periodic calculations. The performance of target-specific AIMNet2 interatomic potentials is illustrated across diverse chemical systems relevant to pharmaceutical, optoelectronic, and agrochemical applications, demonstrating their promise as efficient alternatives to full DFT calculations for routine CSP tasks.

## Graphical abstract

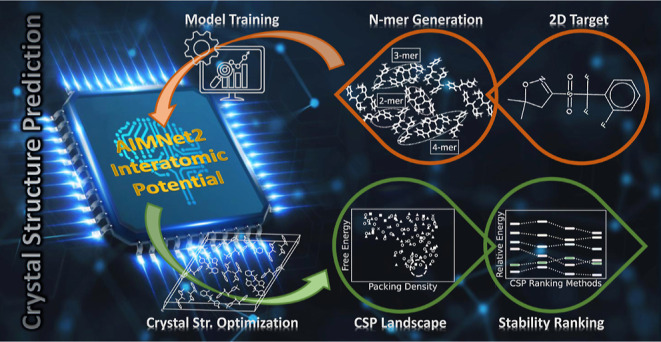

## Introduction

Molecular crystals form through favorable packing of constituent species, stabilized by complex interactions including intramolecular forces, intermolecular dispersion, electrostatics, and polarization. The arrangement of molecules in crystals is crucial for their functionality, as it directly influences the physical and chemical properties of the solid form.^1^ The crystal energy landscape often contains numerous energetically competitive minima within a narrow energy window (typically 2–7 kJ/mol), leading to the ability of a compound to crystallize in multiple solid forms, known as polymorphism. A Cambridge Structural Database (CSD) study has found that nearly 46% of crystal structures containing organic molecules exhibit polymorphism.^2^

The occurrence of polymorphism is influenced by several factors, most commonly by variations in experimental crystallization conditions.^3–5^ At the molecular level, polymorphs may differ in orientation, directional bonding, conformational degrees of freedom, and subtle distortions in bond distances and angles, shaped by the surrounding crystalline environment.^6^ These polymorphic variations significantly impact physicochemical properties despite identical molecular composition, affecting melting points, solubility, density, hardness, optical characteristics, and electrical properties.^7–12^ Understanding polymorphism is thus crucial for applications in pharmaceuticals, organic electronics, and energetic materials, where specific polymorphs can deliver targeted performance improvements.^12–14^ It is often desirable to discover previously unknown, and possibly more stable, polymorphs with improved properties. In fact, commercial CSP studies of 41 pharmaceutical compounds have revealed that 15–45% of currently available small-molecule drugs exist in polymorphic forms other than their most thermodynamically stable counterpart.^15^ This presents significant risks within the pharmaceutical industry, as the use of less stable polymorphs could result in inconsistent drug performance, reduced efficacy, and potential safety concerns, impacting both patient health and the reliability of treatments.

Computational CSP offers an alternative to the time-consuming, expensive and potentially hazardous (in the case of energetic materials) process of experimental polymorph discovery by developing methods that accurately identify molecular crystal polymorphism and determine structure(s) that exhibit desirable properties. CSP involves two key challenges: structure generation across a vast configuration space and accurate stability ranking of candidate structures. Ranking methods must distinguish between polymorphs separated by mere kilojoules per mole (kJ/mol). This demands computationally intensive electronic structure approaches^16^ for modeling molecular crystals, most commonly dispersion-corrected DFT.^17–29^

A good metric of progress in CSP is provided by the results and methods presented in the CCDC blind tests.^30–35^ The CCDC CSP blind test is an internationally recognized scientific challenge that serves as the gold standard for evaluating CSP methods. Participants attempt to predict the 3D crystal structures of “target” molecules solely from their 2D molecular structures, without prior knowledge of their experimentally determined solid forms. The CCDC blind test holds particular importance for the pharmaceutical industry, as predicting polymorphism impacts drug development and formulation. With each iteration, the CSP blind test has featured increasingly complex target molecules, reflecting the evolving capabilities of prediction methods. The seventh CCDC CSP blind test represented a significant step up in complexity, featuring a diverse array of challenging systems including metal–organic complexes, a cocrystal with varying stoichiometry, a salt with a disappearing polymorph, and a large flexible pharmaceutical compound with multiple rotatable bonds. The breadth of these targets illustrates the widespread impact that improving computational CSP workflows can have, with the growing challenge continuing to propel methodological innovation in the field of crystal structure prediction. The seventh CCDC CSP blind test employed a two-phase approach for the first time. In Phase I^36^ (structure generation), researchers submitted a ranked list of predicted structures that they determined to be most likely to match the experimental forms of the given targets. In Phase II^37^ (structure ranking), participants were provided with a common set of structures and were required to relax and rank them based on their stability.

In the first few CSP blind tests, force fields were the only option due to the computing resources available at the time. It was only in the fourth blind test that dispersion-corrected DFT was used on a large scale for final energy minimization and energy ranking of the putative crystal structures.^33^ Although dispersion-corrected DFT has become the community-accepted standard for final structure ranking, force fields are still typically employed for prescreening in the early stages of CSP workflows, and in some cases also in the final ranking process. Recent examples of this include the force fields employed in the sixth and seventh CCDC CSP blind tests,^36–38^ such as COMPASS (2.8),^39^ the DREIDING,^40^ atomic multipole force field, SAPT (DFT) fitted force fields,^41^ XtalPi force fields (XFF)^42^ and tailor-made force field.^43^

The CSP blind tests have shown repeatedly that general-purpose force fields failed to reach the desired accuracy. This has led to the emergence of tailor-made force fields (TMFFs), fit specifically to a particular target molecule using first-principles data. TMFFs can greatly reduce computational expense while still achieving high accuracy.^44,45^ One of the earliest TMFF utilized for CSP, developed by Neumann et al., was effectively applied starting from the fourth CCDC CSP blind test, consistently achieving high success rates.^33,34,38,46–50^ Another example is the CSP procedure developed by Nikhar and Szalewicz,^25^ which begins with a two-dimensional monomer graph and employs a two-body, rigid-monomer ab initio-based force field (aiFF) derived from quantum mechanical calculations for molecular dimers. This approach was shown to work well for a set of selected test systems and was applied in the seventh CCDC CSP blind test. However, the overall results were mixed—particularly poor for highly flexible targets. Many experimental polymorphs remained unidentified, and several that were identified did not meet the 1 Å RMSD_30_ threshold set by CCDC. This was primarily due to the use of only the global-minimum conformer under the rigid-monomer framework for constructing the aiFF, given the high computational cost of the flexible-monomer framework. Polarizable force fields have also been shown to be effective for CSP.^51–53^ However, fitting them requires extensive chemical expertise and laborious parametrization, leading to a high computational cost and significant effort, as is often the case with TMFFs and other system-specific force field approaches. Inconsistent parametrization protocols and variable fidelity have limited the adoption of polarizable force fields.

A promising alternative to both the empirical TMFFs and full DFT calculations are machine-learned interatomic potentials (MLIPs) which can capture complex atomic interactions efficiently while maintaining accuracy comparable to high-level computational methods. MLIPs can achieve near ab initio accuracy while scaling linearly (*O*(*N*)) by using a flexible functional form and training on reference data of energies, forces, and other multimodal atom state information to precisely capture the potential energy surface (PES) of the target chemical system.^54–60^ Recent neural network potentials like AIMNet2 ^61,62^ and MACE-OFF23 ^63^ have demonstrated improved accuracy and transferability for neutral and, in the case of AIMNet2, charged molecules spanning diverse areas of organic and biochemical space. This progress is especially relevant to the CSP problem, where the use of MLIPs and other machine learning techniques has steadily grown in recent years.^64–76^ Another aspect of CSP that can benefit from MLIPs is free energy calculations. Polymorph ranking traditionally relies on static lattice energies at 0 K, neglecting thermal effects.^77^ Hoja and Tkatchenko^78^ demonstrated that accounting for vibrational free energy is essential for accurately describing polymorph stabilities at finite temperatures, as it accounts for entropic effects in the Helmholtz free energy. Previous studies have shown that vibrational and thermal effects contributed an average of 6 and 1.6 kJ/mol in the revised X23 benchmark,^79^ and 5.5 kJ/mol each in a set of 31 energetic materials.^80^ Evaluating these effects with DFT is computationally demanding and can only be performed for a handful of crystal structures. With MLIPs, these calculations become more feasible, even for a large number of crystal structures with many atoms.

MLIPs were used in a blind test for the first time in the seventh CCDC CSP blind test. Our team (Group 16) used system-specific AIMNet2 MLIPs for both the structure generation and ranking phases. In the structure generation phase, MLIPs were used for relaxation and ranking of structures generated by Genarris.^81^ In the ranking phase, MLIPs were used for relaxation, energy evaluation, and free energy corrections. Here, we present a detailed description of our CSP workflow and report a post hoc analysis of our method’s performance. AIMNet2 (second generation atoms-in-molecules network) is a chemically inspired, modular deep neural network molecular potential that combines ML-parametrized short-range and physics-based long-range terms to attain generalizability. This approach captures the necessary physics while drastically reducing computational costs, enabling the screening of millions of candidate crystal structures. Rather than using general-purpose AIMNet2 models in the CSP blind test, system-specific versions (analogous to TMFFs) were fine-tuned and applied for better accuracy. Instead of relying on DFT calculations for periodic crystal structures, we trained AIMNet2 exclusively on molecular cluster (*n*-mer) data, which significantly reduced the computational cost of training data acquisition. In addition to static lattice energy, we incorporated vibrational and thermal effects through the harmonic and quasi-harmonic approximations. The effectiveness of our methodology is highlighted by the results of the seventh CCDC CSP blind test. In the structure generation phase our submission achieved the highest success rate (67%; 4 out of 6 possible structures generated for the targets we submitted) among academic teams, and third overall. In the structure ranking phase two other teams employed MLIPs, based on Gaussian process regression (Group 12) and a transfer learning-based neural network model (Group 15). Our system-specific AIMNet2 potentials were the only successful machine learning submission that consistently aligned with experimental results and DFT. This demonstrates the promise of system-specific AIMNet2 potentials as an efficient alternative to DFT-based methods.

## Methods

### Training and Validation Data Set

To construct a reliable and extensible AIMNet2 interatomic potential for accurate modeling of periodic crystal structures, we assembled an extensive data set of *n*-mer systems. This data set includes a broad distribution of molecular and intermolecular geometries formed mainly by monomers, dimers, trimers and tetramers, with a smaller number of pentamers, hexamers, heptamers and octamers. Our preliminary studies indicated that the higher order set of *n*-mers is necessary for accurately capturing the intermolecular interactions specific to the CSP blind test targets. This section provides an overview of the procedure for generating the training data sets using a large pool of candidate crystal structures and various sampling techniques.

### Conformer Generation

Targets XXVII, XXXI, XXXII and XXXIII were selected from the seventh CCDC CSP blind test. For each CSP blind test target, a dense conformer pool was generated and used as the input for the random crystal structure generator, Genarris.^81^ The approach involved exhaustive enumeration of torsional degrees of freedom based on predefined dihedral angles from a knowledge-based list and the rotatable bond count, utilizing either OpenEye OMEGA^82^ or the RDKit ETKDGv2 method.^83,84^ Subsequently, this set was sampled using geometric and energy considerations. The criteria included generating all flexible ring conformations in a molecule, controlling the enumeration of nonterminal nitrogen atoms, sampling hydrogen locations for amines, and establishing an energy window. Conformers were accepted if the similarity threshold exceeded 0.3 Å and the calculated strain energy was less than the sum of the energy window and the energy of the global minimum conformer, set at 15 kcal/mol (62.76 kJ/mol). Other codes and techniques, such as the BIOVIA Materials Studio (MS) Conformers,^85^ and the Confab algorithm,^86^ were also applied to aid in the systematic generation of diverse low-energy conformers for this task. BIOVIA MS enables the traversal of the potential energy surface (PES) by sampling torsion angles using methods such as systematic grid searching, random sampling, and Boltzmann’s jump, which vary torsion angles to generate multiple conformers and identify low-energy structures. Confab adopts a methodology similar to OpenEye OMEGA, focusing on diversity selection through heavy-atom root-mean-square deviation (RMSD) relative to the already stored conformers. Our conformer generation strategy integrated all the aforementioned methods to enhance coverage of the conformational space and ensure a diverse, low-energy set of initial conformers for each of the target molecule. For the cocrystal salt Target XXXIII, conformers were only generated for the anion fragment. The cation (morpholine) fragment is often locked in a chair conformation that best satisfies its hydrogen bond and packing interactions within the crystal lattice, so no additional morpholine conformations were explored.

### Crystal Structure Generation with Genarris

In the structure generation phase of the seventh CCDC CSP blind test, Genarris^81^ was used to generate crystal structures. These structures were also used for the purpose of extracting *n*-mers to train system-specific AIMNet2 potentials (for the additional targets submitted in the ranking stage, *n*-mers were extracted from the structures provided by the CCDC). Genarris is an open source Python package for generating random homomolecular crystal structures with physical constraints. After selecting a molecular configuration from the conformer pool prepared using the methods described above, Genarris initiates the generation process by estimating the unit cell volume using PyMoVE,^76^ an open source Python package for molecular volume estimation. It then automatically identifies all compatible space groups based on the molecular point group symmetry and the specified number of molecules per unit cell (Z), including those where molecules occupy special Wyckoff positions. Following this, Genarris selects a compatible space group and generates a unit cell within a volume distribution window around the predicted unit cell volume while maintaining space group symmetry. Genarris then places the first molecule randomly in the unit cell. The remaining molecules are positioned using space group symmetry operations. To ensure physically plausible structures, Genarris conducts the following intermolecular distance analysis to ensure that atoms from different molecules are sufficiently spaced apart by assessing the comparison

1 $$d_{A,B}>s_{r}\left(r_{A}+r_{B}\right)$$

where *d*_A,B_ is the distance between atoms A and B from different molecules, *s*_r_ is the specific radius ratio (chosen between 0.7–0.9), and *r*_A_ and *r*_B_ correspond to their respective van der Waals radii. In the seventh CCDC CSP blind test, a development version of Genarris was used. Structures were constructed by picking a molecular geometry uniformly at random from a set of diverse conformers for each generation attempt. In addition, this version of Genarris included a preliminary implementation of the “Rigid Press” algorithm (to be described elsewhere). When Rigid Press is used, the unit cell is initially expanded to facilitate molecule placement and then compressed using a rigid body potential to achieve close packing. During this process, the molecular positions and orientations can change under the *s*_r_ constraint, while their internal geometries remain fixed (or rigid). The preliminary version of Rigid Press that was used for the seventh CCDC CSP blind test did not preserve space group symmetries. The number of generated crystal structures varied depending on the target.

### Molecular Cluster Sampling

Following candidate crystal structure generation, the molecular shell functionality of the CCDC Python Crystal API^87^ combined with a custom-made sampling function, was used to extract molecular clusters from the pool of generated crystal structures. This function yields a set of intermolecular complexes that include atoms within a specified distance cutoff from the central molecule, from which *n*-mers were extracted employing multiple combinations of varying *n* size (*n* = 1–8). *n*-mers were further sampled through short constrained gas phase molecular dynamics simulations in xTB package using the GFNFF-xTB force field^88^ at 300 K for up to 5000 ps to capture out-of-equilibrium conformations. The SHAKE algorithm,^89^ which is used to constrain bond lengths, was disabled. Tethering spring forces were applied to atomic positions using force constants of 0.005 eV/Å^2^. It should be noted that GFNFF-xTB was only used for the purpose of efficient sampling, and was not used for training the AIMNet2 MLIPs. Consequently, the final distribution of *n*-mers ranging from 1 to 8, and the size of the *n*-mer data set, varied for each CSP blind test target based on heuristics. After molecular cluster sampling, single-point DFT calculations were performed using a meta-generalized gradient approximation (*meta*-GGA) functional from the third rung of Jacob’s Ladder,^90^ to obtain the reference energy, atomic forces, partial charges, and dipole data, but only for the monomer set, not the enitre *n*-mer data set.

The final training set was derived from a pool of *n*-mer data (*n* = 1–8) through iterative cycles of active learning (AL), according to the following procedure. We began by calculating energy uncertainties for dimers using four AIMNet2 models, each trained on a small random subset of monomer reference DFT data. The uncertainty σ was defined as $\frac{E_{std}}{\sqrt{N}}$ , where *E*_std_ represents the standard deviation among the predictions from four AIMNet2 models and *N* is the number of atoms in the sample (*n*-mer). If the uncertainty of a dimer exceeded twice the median uncertainty of the entire dimer set (σ > 2 × median­(σ)), the model predictions for that structure were deemed highly uncertain. We then carried out single-point DFT calculations at the same level of theory for these high-uncertainty dimer structures and added them to the initial monomer training pool. The AIMNet2 models were subsequently retrained for the next AL cycle to process trimers. The procedure was then continued iteratively for the remaining *n*-mer sets, one by one. This iterative approach allowed for the identification of the most diverse and chemically informative *n*-mers, thereby improving the accuracy and robustness of the AIMNet2 potentials. The AL procedure was not applied to the cocrystal Target XXXIII due to differences in the types of *n*-mers generated for this system. The final training set for this target comprised charged cation and anion monomers; neutral and charged dimers of the cation and anion; cation–anion fragment pairs; charged cation trimers; combinations of one anion with two cations; and two anions with one cation.

### Model Architecture and Training

In this work, a developmental version of the AIMNet2 ^62^ model was trained to learn atomic contributions to the total interaction energy, atomic forces, and atom-centered partial charges calculated using Hirshfeld partitioning. The models were trained to reproduce DFT-calculated properties and included pairwise C6 and C8 coefficients for explicit long-range atomic-charge dependent London dispersion corrections. All AIMNet2 calculations were performed using the PyTorch framework.^91^ For more information about the model architecture and the computational resources used for training, please refer to Supporting Information and the original AIMNet2 publication.^62^

System-specific AIMNet2 models for the attempted CSP blind test targets were trained on diverse molecular clusters extracted from crystal structures (either generated by Genarris or provided by the CCDC). For these molecular clusters, single-point DFT data (energies, forces, partial charges and dipoles) was calculated, circumventing costly periodic calculations. In the interest of computational efficiency, low accuracy models were first trained using energies, atomic forces, and partial charges derived from the semiempirical tight-binding GFN2-xTB^92^ molecular dynamics trajectories of these clusters. During the preliminary testing stage, it was observed that initializing the AIMNet2 model weights with GFN2-xTB data led to enhanced training stability and faster convergence, in contrast to training directly on data at the DFT-level of theory. Essentially, this pretraining allows the AIMNet2 models to establish rudimentary, albeit lower accuracy, coverage of target-specific potential energy surfaces with a computational cost that is negligible compared to exhaustive DFT. The final set of models underwent training on the DFT reference data for the *n*-mers of each target molecule, obtained through the AL procedure, while excluding all tight-binding initialization data. It is important to highlight that the *n*-mer expansion was truncated based on the convergence of AIMNet2 training; in all cases, using a higher *n* from 5–8 was not mandatory to obtain a well-performing AIMNet2 interatomic potential. Infact, the need for largest *n*-mer size decreases with increasing molecular size, i.e., larger molecules need relatively smaller clusters to effectively capture intermolecular interactions, as their coordination spheres determined by the AIMNet2 cutoff of 5 Å are naturally well populated. In contrast, smaller molecules require larger clusters to ensure sufficient atomic environments within the cutoff.

These trained models were subsequently employed to relax crystal structures with a custom BFGS optimizer implemented in PyTorch, each adhering to a root-mean-squared (RMS) atomic force convergence criterion of 0.001 eV/Å. Only the two-body dispersion term was considered during the optimization process. Once the final optimized crystal geometry was obtained, the three-body dispersion correction was also included. D4-dispersion^93^ corrections and Ewald summation^94^ (with an accuracy of 10^–8^ and a real space cutoff of 15 Å) were incorporated throughout all geometry optimization and thermodynamic calculations to more precisely account for long-range interaction contributions, especially those extending beyond the atomic environment cutoff of the AIMNet2 models. For more information on the training set and the number of crystal structure optimizations conducted for each target, please refer to Table S1 in the Suppporting Information.

### Free Energy Corrections

The relative stability evaluation in the ranking phase of the CSP blind test was performed based on the Gibbs free energy (*G*) using the quasi-harmonic approximation (QHA). The QHA is essentially a collection of harmonic approximations (HAs) executed across several fixed volumes, aiming to extract physical insights about thermally induced volume effects. The finite temperature and vibrational contributions to the Helmholtz free energy (*F*) of a crystal were calculated using the HA and QHA. In the HA, which neglects thermal expansion,^20,22,95^ the Gibbs free energy is defined as

2 $$G=E_{latt}+F_{vib}+pV$$

where *E*_latt_ represents the equilibrium ground-state energy, *F*_vib_ accounts for the vibrational contribution to the Helmholtz free energy (*F*), and *pV* is the pressure–volume work, which is usually negligible at ambient pressure. In the HA, the vibrational free energy per unit cell is calculated from the harmonic phonon frequencies as follows

3 $$F_{vib}=\frac{1}{N_{q}}\sum_{i=1}^{3n}\;\sum_{q}^{N_{q}}\frac{\hbar \omega_{i,q}}{2}+k_{B}T\;\ln\left[1-\exp\left(-\frac{\hbar \omega_{i,q}}{k_{B}T}\right)\right]$$

where *k*_*B*_ is Boltzmann’s constant, *T* is the temperature, *ℏ* is the reduced Planck’s constant, and ω_*i*,**q**_ represents the phonon frequency of mode *i* at wave vector **q**. The sums extend over the 3*n* phonon branches (*n* is the number of atoms in the unit cell) and the *N*_**q**_ sampled **q**-points in the first Brillouin zone. The first term in eq 3 denotes the zero-point energy (ZPE), which corresponds to *F*_*vib*_ at *T* = 0 K, while the second term represents the temperature-dependent component of *F*_*vib*_, commonly referred to as the Kirchhoff term.

The vibrational effects were computed via the HA as implemented in Phonopy,^96^ an open-source Python package designed for phonon calculations at both harmonic and quasi-harmonic levels. Within the Phonopy framework, the phonon density of states and the corresponding thermodynamic properties are computed by constructing the dynamical matrix from force constants. To obtain these force constants, the default finite displacement method was employed, where small atomic displacements (0.005 Å) were systematically applied to atoms in the optimized crystal structure. For each displacement, forces on all atoms were calculated via a single-point calculation using DFT. Supercells were constructed to ensure that the lattice vectors extended at least 10 Å in each direction, minimizing interaction between periodic images and providing accurate force calculations. The phonon frequencies were then obtained by diagonalizing the dynamical matrix at various **q**-points in the Brillouin zone. Using a sufficiently dense **q**-mesh (8 × 8 × 8), the vibrational free energy contribution was calculated with eq 3. The Helmholtz free energy (*F*) was then determined by combining the static lattice energy with these vibrational contributions at the fixed optimized volume, providing a framework for comparing relative stabilities among predicted crystal structures at finite temperatures.

The harmonic phonon theory cannot predict thermal expansion, which can be modeled within the QHA.^97^ In this approach, the effect of volume on the phonon frequencies is taken into account, and the free energy at a given temperature is minimized with respect to volume as follows

4 $$G\left(T,P\right)=\min_{V}\left[U\left(V\right)+F_{vib}\left(T,V\right)+pV\right]$$

The QHA was employed to account for thermal expansion effects on the free energy of crystal structures. Using Phonopy, we extended beyond the standard HA by explicitly calculating vibrational properties at multiple unit cell volumes. After the initial crystal optimization without any applied external pressure, a series of 16 volume-constrained geometry optimizations was conducted for each crystal structure, wherein external isotropic pressure was exerted to modulate the unit cell volume (*V*) within the range of −5% to +15%, spanning from 0.95 to 1.15 times the equilibrium volume at 300 K with uniform increments of 0.0125. For each volume-constrained structure, we calculated the phonon spectrum and corresponding Helmholtz free energy (*F*) at both 0 and 300 K, using a well-converged 8 × 8 × 8 reciprocal space sampling mesh. The free energy versus volume (*F* – *V*) data were then fitted to the Birch–Murnaghan equation of state^98^ to determine the minimum Helmholtz free energy and corresponding volume at each temperature of interest. The Gibbs free energy (*G*) was obtained as the minimum value along this *F* – *V* curve, effectively capturing anharmonic contributions arising from thermal expansion. Temperature-dependent properties, including the constant pressure heat capacity (*C*_p_), were subsequently derived from the temperature dependence of the Gibbs free energy according to the following relation

5 $$C_{p}\left(T,p\right)=-T\frac{\partial^{2}G\left(T,p\right)}{\partial T^{2}}$$

During the second phase of the CCDC CSP blind test, free energy ranking was performed by evaluating the Helmholtz free energy (*F*), with vibrational contributions and thermal expansion effects included. It is worth emphasizing that the efficiency of the AIMNet2 models supports sufficiently dense volume sampling without a significant computational overhead, with the final choice of 16 samples being informed by an accuracy-volume scan density analysis performed during initial testing. Each expanded or contracted crystal structure underwent a fixed-volume variable-cell geometry optimization using a custom BFGS optimizer implemented in PyTorch. The optimization convergence criteria are outlined in the computational details section in Supporting Information. For each optimized geometry along the volume scan, a HA calculation was performed using Phonopy. Unlike the typical and computationally expensive finite displacement approach with DFT, atomic force constants were obtained directly from the analytical Hessian, derived through automatic differentiation applied to the AIMNet2 model outputs. Hence, this approach offers a significant advantage in conducting QHA/HA calculations with MLIPs, as it is more efficient than its numerical counterpart and avoids potential errors associated with choosing improper displacement sizes.

### Density Functional Theory Calculations

In the molecular cluster (*n*-mer) reference data generation process, we utilized the B97M^99^ functional with the Karlsruhe triple-ζ basis set featuring two sets of polarization functions (def2-TZVPP)^100,101^ in the gas phase to obtain data for training each system specific MLIP. All single-point energy calculations were carried out using the Orca 5.0.3 software.^102^ Additionally, molecular dipole–dipole dispersion coefficients and polarizabilities were included using the DFT-D4 python package^93^ based on the Axilrod–Teller–Muto (ATM) formula.

All post hoc DFT calculations were conducted using the FHI-aims electronic structure code^103^ (version 200112). A *k_grid* of size *n* × *a* ≥ 25, where *n* is the number of *k*-points and *a* represents the length of the lattice vector, was utilized for all calculations.^20,21,27^ The generalized gradient approximation of Perdew, Burke, and Ernzerhof (PBE)^104^ was paired with the many-body dispersion (MBD)^105^ method. Geometry relaxations, lattice energy evaluations, and free energy corrections were performed using *lower*-level settings, which correspond to the *tier 1* basis sets and *light* species defaults of FHI-aims. The experimental structures were further reoptimized with PBE + MBD using *higher*-level settings, which correspond to the *tier 2* basis sets and *tight* species defaults of FHI-aims. These structures were reranked based on single-point energy evaluations using the PBE-based hybrid functional, PBE0,^106^ paired with the MBD method.

## Results and Discussion

The seventh CCDC CSP blind test was conducted in two phases to highlight the two primary aspects of any CSP workflow: structure generation and structure ranking. In Phase I (structure generation), a list of 1500 generated crystal structures ranked by the lattice energy was submitted for each of the attempted target compounds. This list was later examined by CCDC organizers for matches with the experimentally observed polymorphs. In Phase II (structure ranking), CCDC provided participants with a set of either 100 or 500 selected crystal structures (depending on the target), which needed to be relaxed and ranked according to their relative stability. The lists included the experimentally observed polymorphs as well as putative structures drawn from a global data set comprising all submissions from the structure generation phase.

Figure 1 shows a high-level overview of our MLIP-based CSP workflow. The approach involves generating a large number of random crystal structures, conducting geometry optimization with system-specific AIMNet2 potentials, and subsequently choosing structures with low lattice energies. The pipeline begins with a conformational search, as described above, proceeds to crystal structure generation with Genarris, and subsequently to *n*-mer sampling and AIMNet2 training by active learning. Once the system-specific AIMNet2 potentials are trained, relaxation can be performed efficiently for millions of structures, producing an energy landscape. A smaller subset of the structures predicted to be most stable by AIMNet2 can be reranked with more accurate methods and/or with free energy corrections.

**Figure 1:**
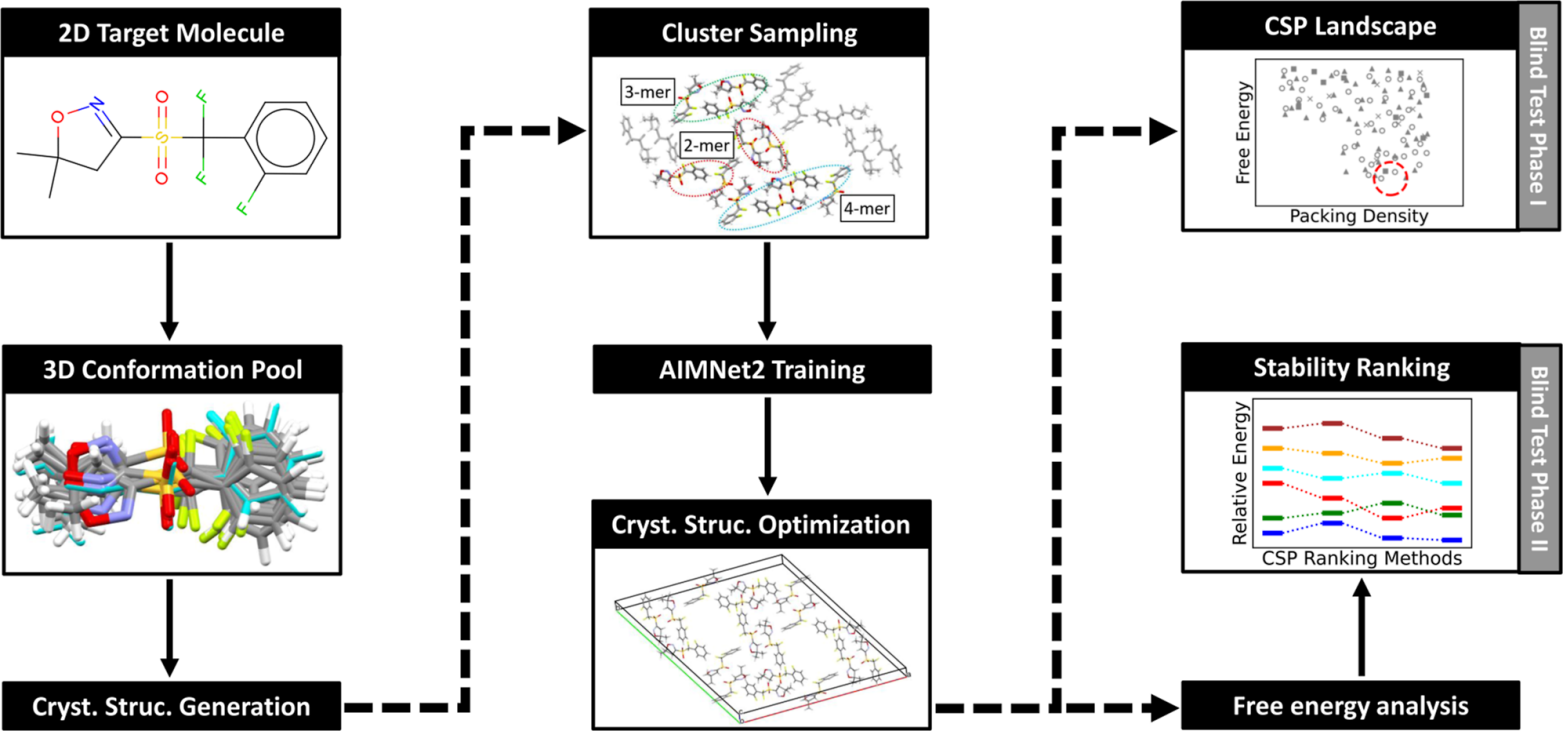
Schematic diagram of the CSP workflow, which was used in the structure generation phase of the seventh CCDC CSP blind test.

The selected CSP blind test Targets XXVII, XXXI, XXXII and XXXIII, shown in Figure 2, can be categorized into optoelectronic materials, pharmaceutical or agrochemical-like compounds, and molecules with diverse chemistry and industrial significance. Molecule XXVII, [(2,3-diiodopentacene-6,13-diyl)­bis­(ethyne-2,1-diyl)]­bis­(triisopropylsilane) is a silicon- and iodine-substituted triisopropylsilyl (TIPS) pentacene derivative intended for use in organic electronics.^107,108^ Molecule XXXI, 3-((difluoro-(2-fluorophenyl)­methyl)­sulfonyl)-5,5-dimethyl-2l2-isoxazolidine, is a simple agrochemical with three rotatable bonds. Molecule XXXII, or (*N*-(3-[2-(difluoromethoxy)-5-(methylthio)­phenyl]-1-[2-(4-morpholinopiperidin-1-yl)-2-oxoethyl]- 1*H*-pyrazol-4-yl)­pyrazolo-[1,5-*a*]­pyrimidine-3-carboxamide) is a large pharmaceutical compound with 11 rotatable bonds. Lastly, Molecule XXXIII is a 1:1 morpholine salt of 4-amino-*N*-(5-methylisoxazol-3-yl)-benzenesulfonamide (commercially known as Sulfamethoxazole). CSP for Target XXVII was challenging because of the conformationally flexible substituents, leading to a very large conformational space. Target XXXI was challenging, owing to the rotational disorder of the *ortho*-fluorophenyl ring and difluoromethyl group, and fluorine atoms susceptible to positional disorder. This required screening millions of trial structures for each of these targets. This was enabled thanks to the computational efficiency of the AIMNet2 models, which are faster than DFT by a factor of over a thousand. In the structure ranking phase, the efficiency of AIMNet2 facilitated free energy evaluations for hundreds of structures, including structures with large unit cells (Target XXXII). It is also noteworthy that AIMNet2 can handle charged species, such as the salt Target XXXIII, and elements such as Si, I (Target XXVII), S and F (Targets XXXI, XXXII), which other MLIPs may not support or struggle with.

**Figure 2:**
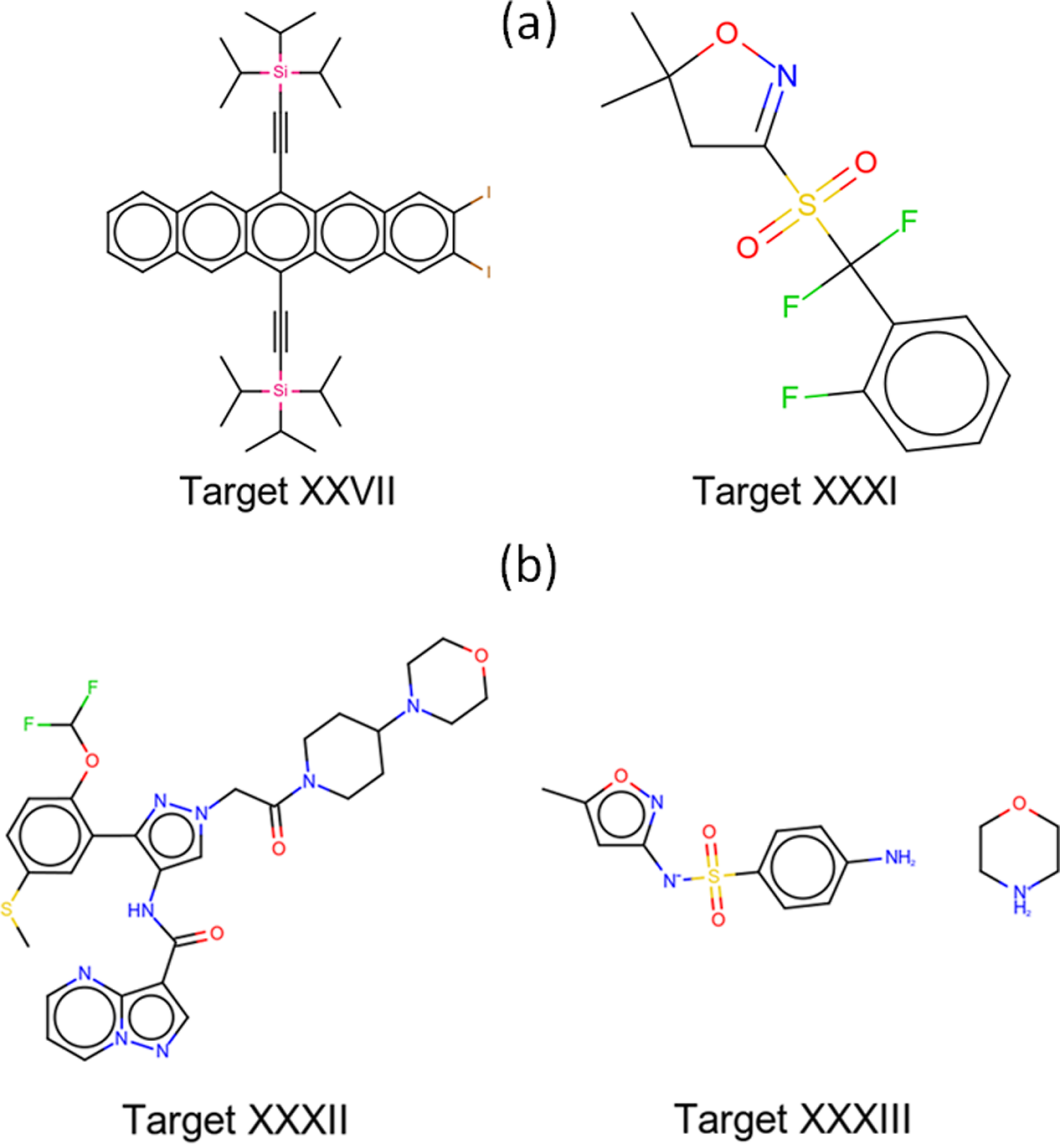
(a) Selected targets attempted in both Phase I and II, and (b) additional targets attempted only in Phase II of the seventh CCDC CSP blind test.

### CSP Blind Test Phase I: Structure Generation

For target XXVII, AIMNet2 models were trained within 8 AL iterations on a total of 9.5 × 10^4^ *n*-mers, comprising 13 × 10^3^ monomers, 75 × 10^3^ dimers, and 7 × 10^3^ trimers. An ensemble of 1.8 × 10^4^ conformers was used to generate 5.0 × 10^6^ crystal structures with *Z* = 2, and 4 with Genarris. After geometry optimization with AIMNet2, the structures were symmetrized using Spglib,^109^ with a distance tolerance in Cartesian coordinates (symprec) of 0.2 Å. Duplicate structures were then removed using pymatgen’s^110^ StructureMatcher, applying a site tolerance of 0.2 Å along with default fractional length (lattice) and angle tolerances (stol = 0.2 Å, ltol = 0.2 Å, angle_tol = 5°).

For Target XXXI, AIMNet2 models were trained within 4 AL iterations on a total of 22.13 × 10^4^ *n*-mers, comprising 6.3 × 10^3^ monomers, 125 × 10^3^ dimers, 76 × 10^3^ trimers, and 14 × 10^3^ tetramers. An ensemble of 658 conformers was used to generate 3.5 × 10^6^ crystal structures with *Z* = 2, 4, and 8 with Genarris. Following geometry optimization with AIMNet2, the structures were symmetrized, and duplicates were removed using the same settings as those applied to Target XXVII. A small subset of the top-ranked AIMNet2 structures (from a total of 1.7 × 10^6^ structures after removing duplicates) was selected for DFT calculations and reoptimized with FHI-aims using PBE + MBD method and *lower*-level settings. The final submission included a combination of 1006 structures optimized by DFT and 494 structures optimized by AIMNet2, labeled by the prefixes “aimnet” or “dft”, respectively.

The known crystal structure of Target XXVII, Form A, crystallizes in the *P*1̅ space group with a single molecule in the asymmetric unit. An initial structure was obtained at 90 K before the blind test began and later rerefined as having I/Br disorder as a result of Br contamination during the synthesis (CSD refcode: *XIGYUL*). Eventually, the structure was redetermined from pure material with diffraction data collected at 100 K (CSD refcode: *XIFZOF01*). It was found that the bromine impurity did not significantly impact the crystal structure.

The experimental form of Target XXVII was successfully generated during the structure generation phase. In Figure 3a, the AIMNet2-based relative lattice energy is shown as a function of crystal density for the 1500 submitted structures. One structure is found to be significantly lower in energy than the others, although it is not the closest to the experimental form. The experimental form (90 K) is ranked much higher in energy (ranked 297/1500), nearly 13 kJ/mol above the minimum energy structure.

**Figure 3:**
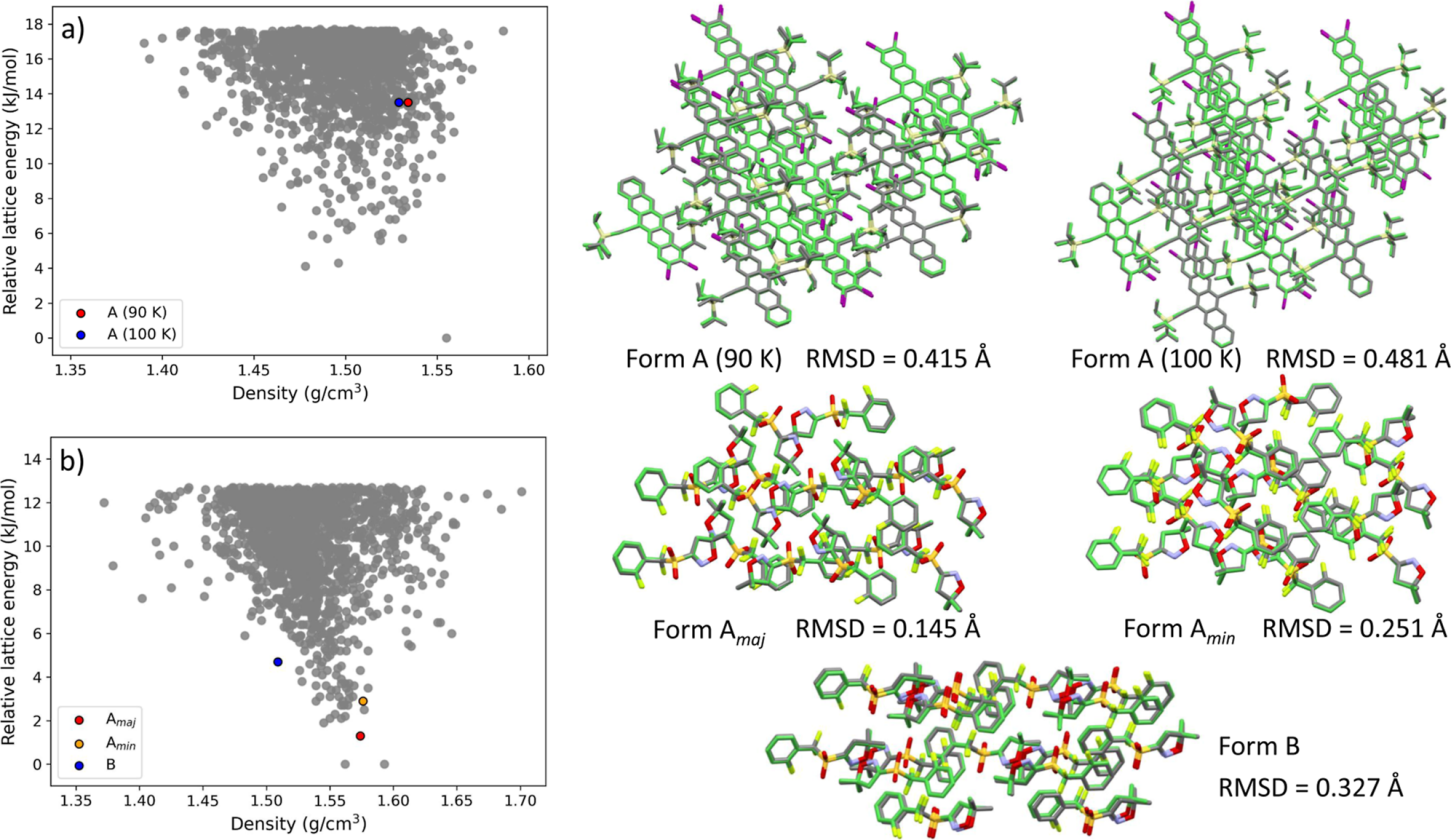
Results of the structure generation phase: Relative lattice energy vs density landscapes obtained with AIMNet2 for (a) Target XXVII and (b) Target XXXI. Overlays of the structures relaxed with AIMNet2 on top of the respective experimental structures (colored by element) are also shown. Hydrogen atoms were omitted for clarity.

For Target XXXI, three crystal structures are known, labeled as Forms A, B, and C. This target was the most polymorphic among those included in the seventh CCDC CSP blind test, which offered significant challenges in both the structure generation and structure ranking phases. Form A (CSD refcode: *ZEHFUR02*) crystallizes in space group *P*2_1_/*c* with one molecule in the asymmetric unit. The *ortho*-fluorophenyl ring is disordered over two sites, designated Form A_maj_ and A_min_, in a 60:40 ratio. Form B (CSD refcode: *ZEHFUR*) also crystallizes in space group *P*2_1_/*c* with one molecule in the asymmetric unit. Form C (CSD refcode: *ZEHFUR01*) crystallize in space group *R*3̅. It is a porous structure with void channels, whose formation is likely promoted and stabilized by the presence of solvent molecules. It has been experimentally determined that Form B is more stable than Form A at 346 K and below.^36^

Figure 3b shows the AIMNet2-based relative lattice energy vs crystal density plot for the 1500 submitted structures of Target XXXI. Three of the four known experimental structures were generated, A_maj_, A_min_, and B. The solvent stabilized Form C, which is expected to be relatively high in energy due to its porous nature, was not generated by any of the participants. It is generally challenging to predict the occurrence of porous molecular crystal structures that are relatively high in energy.^111^ Here, the experimental forms are positioned in the relatively low-energy region of the landscape compared to Target XXVII. The A_maj_ form (ranked 3/1500) is correctly ranked as more stable than the A_min_ form (ranked 15/1500). However, Form B (ranked 46/1500) is incorrectly ranked as less stable than Form A (see further discussion of stability ranking below). Only two roughly degenerate structures were found to have a lower energy than Form A_maj_ by less than 2 kJ/mol. Upon further examination, we found that these two structures share the same space group, *P*2_1_/*c*, as Form A_maj_, but differ in their unit cell orientations and molecular packing arrangements compared to Form A_maj_. Additionally, a separation of ∼1 kJ/mol exists between the Forms A_min_ and A_maj_, with a further gap of about 1–2 kJ/mol between the Forms A_min_ and B.

For both targets the energy landscapes obtained from our CSP workflow manifest the so-called overprediction problem.^112^ CSP workflows tend to significantly overestimate the number of possible crystal structures because structures that appear to be distinct energy minima at 0 K may correspond to the same structure at finite temperature. In particular, for Target XXVII, whose crystal structure exhibits disorder in the TIPS groups, multiple local minima with different TIPS group conformations coalesce into one disordered structure at finite temperature. In such cases, our workflow could benefit from further clustering and coarse-graining of the energy landscape in the future.

### CSP Blind Test Phase II: Structure Ranking

In the structure ranking phase, we received 100 structures of Target XXVII. The experimental structure of Form A determined at 90 K was absent from this set. Instead, four structures that exhibited the same core packing but varied in their isopropyl conformations were included. These were numbered 28, 38, 59, and 61 and had RMSD_30_ of 0.53, 0.80, 0.83, and 0.57 Å, respectively. For Target XXXI, three experimental forms were included in the list of 100 structures: A_maj_ (structure 98), A_min_ (structure 1), and B (structure 25). Form C, identified as a channel-type solvate with unresolved solvent, was excluded from the ranking exercise, but is included in our analysis. For Target XXXII, a list of 500 structures was provided, which included the two polymorphs, whose structures were determined at 90 K. Form A (CSD refcode: *JEKVII*) crystallizes in the *P*1̅ space group, with one molecule in the asymmetric unit and rotational disorder of the difluoromethyl group (structure 317). Form B (CSD refcode: *JEKVII01*) also crystallizes in the *P*1̅ space group, but with two molecules in the asymmetric unit (structure 232). For Target XXXIII, we received a list of 500 structures, including its two known forms. Both Form A (CSD refcode: *ZEGWAN*) and Form B (CSD refcode: *ZEGWAN01*) contain one formula unit in the asymmetric unit. In both forms, proton transfer from the sulfonamide nitrogen to morpholine results in the formation of a salt. Form A (structure 233), determined at 296 K, crystallizes in the monoclinic space group *C*2/*c* with a tetrameric packing motif. Form B (structure 452), determined at 297 K, crystallizes in the orthorhombic space group *Pna*2_1_. Its crystal structure consists of zigzag chains of sulfamethoxazole linked via morpholine molecules.

For all four targets, the structures we received from the CCDC were relaxed and ranked with system-specific AIMNet2 potentials. Further free energy calculations were also performed using AIMNet2. Our submission consisted of two lists, one ranked by lattice energy and the other by free energy. For Target XXVII and Target XXXI, we used the AIMNet2 potentials already trained during the first phase of the blind test. For Target XXXII and Target XXXIII, new AIMNet2 potentials were trained for the ranking phase. For Target XXXII, AIMNet2 potentials were trained within 4 AL iterations on a total of 0.62 × 10^4^ *n*-mers, comprising 1.03 × 10^3^ monomers, 2.68 × 10^3^ dimers, and 2.52 × 10^3^ trimers. For Target XXXIII, AIMNet2 potentials were trained on a total of 2.25 × 10^4^ *n*-mers, comprising 1.59 × 10^3^ monomers, 7.37 × 10^3^ dimers, and 13.58 × 10^3^ trimers without using AL. Fewer *n*-mers were used during the ranking phase than during structure generation, as the latter required an interatomic potential capable of broadly exploring the crystal energy landscape. In contrast, the *n*-mers for the ranking phase were extracted from a fixed set of structures provided by CCDC, resulting in a smaller and more focused training data set. Free energy calculations within the QHA were performed using AIMNet2 on a set of 16 distinct volume-constrained structures at 300 K, which was used to determine the Helmholtz free energy (*F*) as a function of volume (*V*). This amounted to a total of 1600 or 8000 calculations for each target, depending on the number of structures provided by the CCDC (100 or 500). Notably, AIMNet2 enabled these QHA calculations at a speed exceeding that of DFT by orders of magnitude. In addition, for Target XXVII, Target XXXI, and Target XXXIII, we relaxed and ranked the structures provided by the CCDC using PBE + MBD with *lower*-level settings. These DFT calculations were performed for validation purposes and were not included in our blind test submission.

Figure 4 presents the results of the DFT validation for Target XXVII, Target XXXI, and Target XXXIII. The performance of AIMNet2 is assessed based on two metrics: the accuracy of the relaxed geometry, evaluated by comparing the unit cell volume and RMSD_30_ to the DFT reference, and the relative energy ranking. Overall, AIMNet2 tends to overestimate the volume compared to PBE + MBD. This could be attributed to differences in the DFT functionals (B97 M vs PBE) and dispersion correction schemes (D4 vs MBD) used in the AIMNet2 training and the reference assessment method. The best relaxation performance is obtained for Target XXXI, for which the RMSD_30_ is below 0.4 Å for the vast majority of structures. The performance of AIMNet2 for Target XXXIII is somewhat worse with the RMSD_30_ histogram peaked around 0.4 Å. For Target XXVII most structures have RMSD_30_ values above 0.4 Å. This is also reflected in the more significant unit cell volume overestimation for this target.

**Figure 4:**
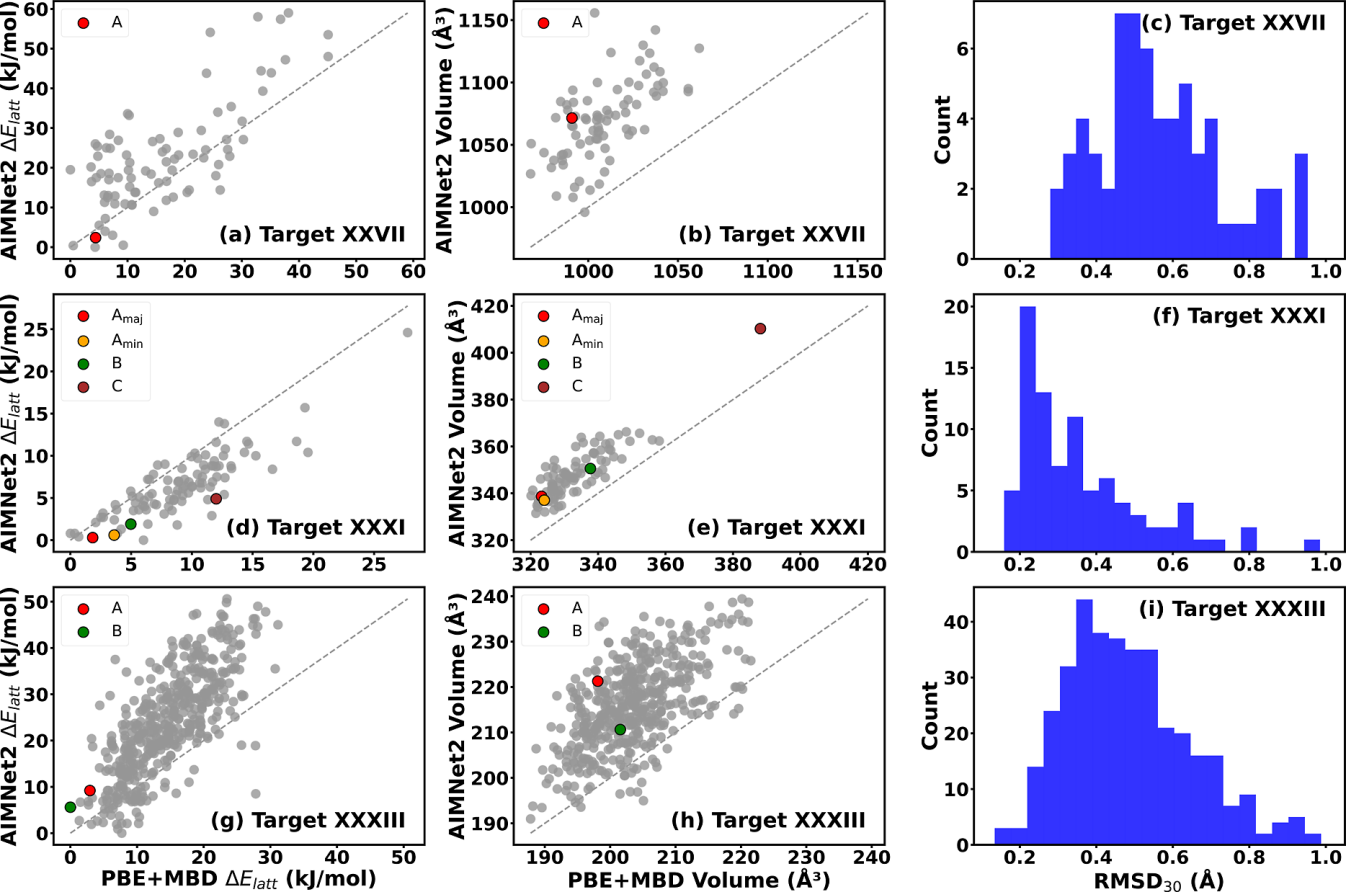
Assessment of the performance of AIMNet2 for the crystal structures provided in the ranking phase of the seventh CCDC CSP blind test. Comparison of the relative energy ranking, unit cell volume, and RMSD_30_ between AIMNet2- and DFT-relaxed structures for Targets XXVII (top row, a–c), XXXI (middle row, d–f), and XXXIII (bottom row, g–i).

For all three targets, the relative energy ranking obtained with AIMNet2 is overall reasonably consistent with DFT and the experimentally observed structures are ranked relatively low in energy (with the exception of Form C of Target XXXI). The relative energies obtained for Target XXXI match DFT more closely than for Target XXXIII and Target XXVII. The lower performance observed for Target XXVII is likely due to its larger molecular and unit cell size, which increases the system’s complexity. Moreover, the presence of highly flexible TIPS side groups introduces additional interaction variability, requiring more extensive training data for the interatomic potential to achieve higher accuracy. The AIMNet2 model achieved a mean absolute error (MAE) of 2.88 kJ/mol and a root-mean-square error (RMSE) of 3.53 kJ/mol for Target XXXI when compared to the resulting DFT values for relative lattice energies. For Target XXXIII, the model yielded an MAE of 10.08 kJ/mol and an RMSE of 12.08 kJ/mol, while for Target XXVII, the MAE and RMSE were 8.66 and 11.16 kJ/mol, respectively.

Table 1 summarizes the performance of target-specific AIMNet2 models and the medium variant of the MACE-OFF23 ^63^ pretrained transferable organic force field, denoted as MACE-OFF23­(M), with respect to DFT reference values. The MACE-OFF23 medium model was selected for comparison because it matches the AIMNet2 models from both the phases in terms of size, specifically the total number of model parameters involved. As MACE-OFF23­(M) does not support the chemical element Si, results for Target XXVII are unavailable. Additionally, only relative energy rankings were computed using MACE-OFF23­(M) without retaining optimized geometries; hence, unit cell volume comparisons for all three targets have not been reported at this time. Overall, the custom-fitted AIMNet2 models consistently outperform the off-the-shelf MACE-OFF23­(M) model, exhibiting lower MAE and RMSE values, along with higher Kendall rank correlation coefficients. Specifically, for Target XXXI, AIMNet2 achieves lower MAE by 42.3% and lower RMSE by 40.1%; for Target XXXIII, MAE and RMSE reduce by 57.9% and 55.1%, respectively, relative to MACE-OFF23­(M)—demonstrating much better agreement with DFT. This improved performance can be attributed to the target-specific training of the AIMNet2 models.

**Table 1:** Comparison of the Performance of AIMNet2 and MACE-OFF23­(M) against DFT Reference for the Crystal Structures Provided in the Ranking Phase of the Seventh CCDC CSP Blind Test^a^

target	model	**RMSD**_30_ (Å)	Matched (%)	MAE (kJ/mol)	RMSE (kJ/mol)	Kendall rank
XXVII	AIMNet2	0.56 ± 0.17	76	8.66	11.16	0.44
	MACE-OFF23(M)	N/A	N/A	N/A	N/A	N/A
XXXI	AIMNet2	0.36 ± 0.16	88	**2.88**	**3.53**	**0.63**
	MACE-OFF23(M)			4.99	5.89	0.52
XXXIII	AIMNet2	0.48 ± 0.16	78	**10.08**	**12.08**	**0.57**
	MACE-OFF23(M)			23.94	26.84	0.48

a The best results are highlighted in bold. Lower MAE and RMSE, and higher Kendall rank correlation coefficient, indicate better agreement with the reference.

Table 2 presents further analysis comparing the results of geometry relaxation with AIMNet2 to PBE + MBD with *lower*-level and *higher*-level settings for the experimental forms of all four targets. We find that switching from *lower*-level to *higher*-level settings in the PBE + MBD calculations only leads to a slight reduction in the RMSD. In most cases, the system-specific AIMNet2 potentials deliver an excellent performance with RMSD values on par with DFT.

**Table 2:** Comparison of the Structures Optimized with AIMNet2 and DFT (PBE + MBD with *Lower*-Level and *Higher*-Level Settings) for the Polymorphs of Targets XXVII, XXXI, XXXII, and XXXIII^a^

		RMSD_30_ (Å)		
target	experimental Form	AIMNet2	PBE + MBD (*lower*-level)	PBE + MBD (*higher*-level)
XXVII	A	0.574	0.629	0.582
XXXI	A_maj_	0.214	0.145	0.107
	A_min_	0.240	0.207	0.226
	B	0.479	0.221	0.203
	C	0.178	0.133	0.123
XXXII	A	0.231	0.201	0.175
	B	0.353	0.300	0.296
XXXIII	A	0.359	0.182	0.151
	B	0.196	0.190	0.192

a The RMSD_30_ values are with respect to the experimentally determined structures.

Upon heating, molecular crystals undergo considerable expansion from their so-called electronic volume (*V*_el_) at 0 K. This expansion can be split into the contribution of the zero-point vibrational energy at 0 K (*V*_ZPVE_) and the contribution of thermal vibrational motion at room temperature (*V*_RT_).^20,27,113^ Both effects can be evaluated within the QHA, as described above. Figure 5 presents the percent error with respect to the experimental unit cell volume, obtained using PBE + MBD with *lower*-level settings when vibrational and thermal effects are taken into account, and with AIMNet2 at 0 K (*V*_AIMNet2_), for the polymorphs of Target XXXI, Target XXXII, and Target XXXIII. The PBE + MBD electronic volume at 0 K is underestimated compared to the experimental volume in all cases except the solvent-stabilized Form C of Target XXXI. Adding the ZPVE contribution at 0 K results in some volume expansion. For Forms A_maj_, A_min_, and C of Target XXXI, this leads to a slightly overestimated volume compared to experiment, whereas for Form B, and all forms of Targets XXXII and XXXIII, *V*_ZPVE_ is still underestimated. Considering vibrational effects and thermal expansion at 300 K leads to further volume expansion, which is particularly significant for Forms A_maj_, A_min_, and C of Target XXXI, resulting in considerable overestimation of the experimental volume. For Form B of Target XXXI and the two forms of Target XXXII, *V*_RT_ is slightly overestimated, whereas for the two forms of Target XXXIII, *V*_RT_ is slightly underestimated. The unit cell volume obtained with AIMNet2 is somewhat overestimated for all forms of Target XXXI and Form A of Target XXXIII, but slightly underestimated for the two forms of Target XXXII and Form B of Target XXXIII.

**Figure 5:**
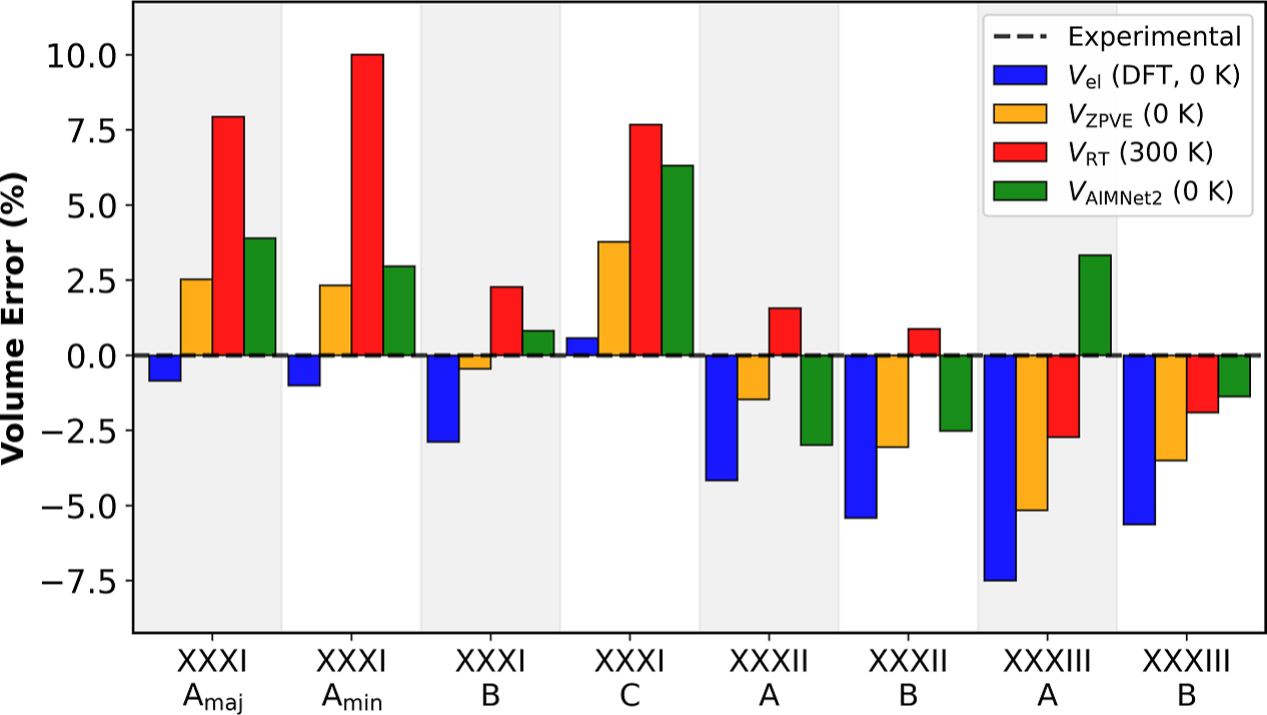
Comparison of the unit cell volume obtained with AIMNet2 at 0 K (*V*_AIMNet2_) to that obtained with DFT at 0 K (*V*_el_), with a zero-point vibrational energy correction (*V*_ZPVE_), and at room temperature with free energy corrections including thermal expansion within the QHA (*V*_RT_), for the polymorphs of Target XXXI, Target XXXII, and Target XXXIII. All calculations except *V*_AIMNet2_ (0 K) employ PBE + MBD with *lower*-level settings.

In addition to the unit cell volume, accounting for vibrational and thermal contributions can affect the relative stability of molecular crystal structures. For Target XXVII, although the experimental structure was ranked relatively high in energy out of the 1500 putative structures we submitted in the structure generation phase (see Figure 3a), out of the list of 100 structures we received in the ranking phase, the experimental structure was ranked by AIMNet2 as the most stable both in terms of lattice energy and free energy.^37^ In comparison, the two other teams that used MLIPs, Group 12 and Group 15 ranked the experimental structure as #25 (lattice energy) and #56 (free energy), respectively. Seven teams (Groups 3, 5, 9, 10, 11, 20, 22) submitted ranking entries for Target XXVII using various DFT methods. The DFT ranking of the experimental structure ranged from #1 to #7, depending on the method used and whether free energy corrections were included. Our DFT lattice energy ranking obtained using PBE + MBD and *lower*-level settings (see Figure 4a) is #7. Hence, the DFT results presented here are consistent with the DFT results reported in the blind test.

For Target XXXI, the experimentally determined order of stability is B > A > C at both 0 and 300 K.^37^ Of the list provided by the CCDC, our team ranked Form A_maj_ as #2 based on AIMNet2 lattice energy and #3 based on free energy. Form A_min_ was ranked #4 based on lattice energy and #18 based on free energy. Form B was ranked as #10 based on both lattice energy and free energy. All three forms were within an energy window of less than 5 kJ/mol of the global minimum. The two other teams that employed MLIPs ranked the Forms A_maj_, A_min_, and B higher above their respective global minima as #33, #38, and #12 (Group 12, lattice energy) and #18, #13, and #12 (Group 15, free energy). Furthermore, during the analysis for Table 1, we observed that MACE-OFF23­(M) ranked the Forms A_maj_, A_min_, and B as #9, #45, and #6, respectively, based on lattice energy. Ten teams submitted ranking entries for Target XXXI using various DFT methods (Groups 2, 3, 4, 5, 9, 10, 11, 14, 20, 22). All DFT methods predicted Forms A_maj_, A_min_, and B to be within 5.7 kJ/mol of the global minimum, although the order of stability varied. In most cases, Form B was ranked higher than Forms A_maj_ and A_min_. Group 20 ranked Form B as the most stable of the three known forms and #6 overall based on free energy. Only two of these groups ranked Form B as the global minimum, Group 10 based on lattice energy (and #3 based on free energy) and Group 3, based on free energy (and #6 based on lattice energy).

Figure 6a shows the relative energy ranking of the polymorphs of Target XXXI, obtained using AIMNet2, compared to different DFT methods, with and without vibrational and thermal corrections. Form C is always ranked as the least stable and Form A_maj_ is always ranked as the most stable. Based on lattice energy, Form B is ranked as less stable than Forms A_maj_ and A_min_ using PBE + MBD, PBE0 + MBD, and AIMNet2. As shown in Figure 4d, PBE + MBD with *lower*-level settings ranks Form A_maj_ as #4, Form A_min_ as #9, Form B as #16, and Form C as #79. When the zero-point vibrational energy contribution is added to the PBE0 + MBD lattice energy, Form B is still ranked as less stable than Form A, but closer in energy to Form A_min_. When the free energy at room temperature is calculated within the QHA, Form B becomes more stable than Form A_min_ using both PBE + MBD and AIMNet2. Thus, the ranking obtained with AIMNet2 is consistent with the DFT results presented here, which are consistent with some of the DFT results submitted by other groups in the ranking phase of the blind test.

**Figure 6:**
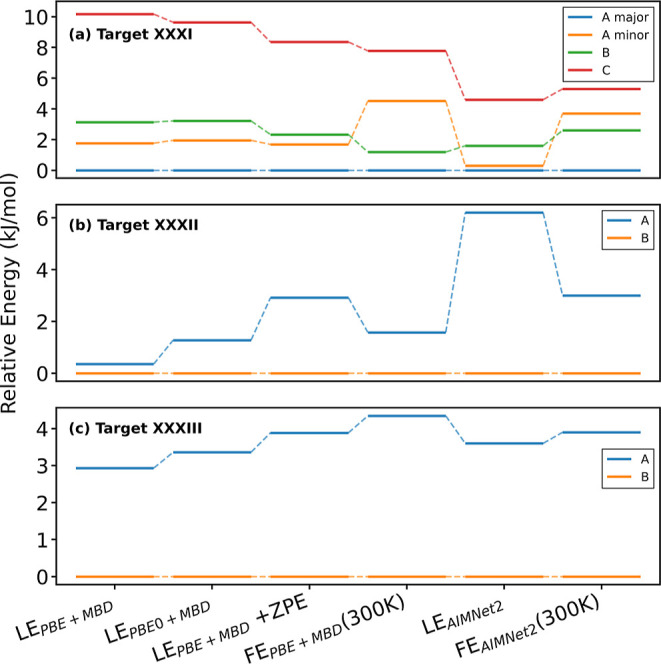
Relative energy ranking of the polymorphs of (a) Target XXXI, (b) Target XXXII, and (c) Target XXXIII obtained based on lattice energies (LE) calculated with PBE + MBD and PBE0 + MBD, PBE + MBD with zero-point energy (ZPE) corrections, and PBE + MBD free energies (FE) calculated using the QHA at 300 K, compared to AIMNet2 lattice energy and free energy.

For Target XXXII, Form B was experimentally found to be more stable than Form A.^37^ This target proved particularly challenging in the ranking stage. All blind test participants ranked the two experimentally observed forms relatively high (over 3 kJ/mol) above the global minimum out of the list of structures provided by the CCDC. Our team was one of only four groups that correctly ranked Form B as more stable than Form A. Based on AIMNet2 lattice energy, Form B was ranked as #3, approximately 6.3 kJ/mol above the global minimum and Form A was ranked as #41, around 12.4 kJ/mol above the global minimum. In this case, applying vibrational and thermal free energy corrections did not improve the results with Form B ranked as #15, approximately 13 kJ/mol above the global minimum, and Form A ranked as #38, around 15.9 kJ/mol above the global minimum. In comparison, the MLIP of Group 12 ranked Form B as #129 and Form A as #490, based on lattice energy. The MLIP of Group 15 failed to relax the structure of Form B and their optimized geometry no longer matched the experimental structure. They ranked Form A as #18 based on free energy.

Eight teams submitted ranking entries for Target XXXII using various DFT methods (Groups 2, 3, 4, 5, 10, 11, 20, 22). All of them ranked Form A as more stable than Form B. Figure 6b shows the relative energy ranking of the polymorphs of Target XXXII, obtained using AIMNet2, compared to different DFT methods, with and without vibrational and thermal corrections. All the methods used here consistently rank Form B as more stable than Form A. With PBE + MBD and *lower*-level settings there is a very small energy difference of 0.355 kJ/mol between the lattice energies of the two forms. The energy difference increases when switching to PBE0 + MBD and when applying vibrational and thermal free energy corrections. Of the teams that submitted DFT rankings in the blind test, Group 10 used PBE0 + MBD to compute lattice energies, however they ranked Form B (#30) as less stable than Form A (#13). It is possible that they may have employed an alternative implementation or different convergence settings.

For the cocrystal Target XXXIII, Form B was experimentally found to be more stable than Form A, which is a metastable disappearing polymorph.^37^ For this target, eight teams submitted ranking entries for using various DFT methods (Groups 3, 4, 5, 9, 10, 11, 20, 22). All correctly ranked Form B as more stable than Form A and all but one (Group 4) ranked Form B as the global minimum. Our PBE + MBD results, shown in Figure 4g, are consistent with the DFT results reported in the blind test, ranking Form B as the global minimum, and Form A as #6, 2.9 kJ/mol above the global minimum. AIMNet2 correctly ranked Form B as more stable than Form A, however both forms were ranked relatively high above the global minimum. Form B was ranked #20 based on both lattice energy and free energy, about 5 and 8.1 kJ/mol above the global minimum. Form A was ranked as #60 based on lattice energy, approximately 8.5 kJ/mol above the global minimum and #56 based on free energy, around 10.7 kJ/mol above the global minimum. The MLIPs used by other teams performed poorly for this target. Group 12 ranked Form A as #90 and Form B as #470. The MLIP used by Group 15 failed to relax Form A and its optimized structure no longer matched the experimental structure, whereas Form B was ranked as #288 based on free energy. Additionally, MACE-OFF23­(M) ranked the Form A as #167 and Form B as #204 based on lattice energy. Figure 6c shows the relative energy ranking of the polymorphs of Target XXXIII, obtained using AIMNet2, compared to different DFT methods, with and without vibrational and thermal corrections. All the methods used here consistently rank Form B as more stable than Form A. Switching from PBE to PBE0 and adding vibrational and thermal corrections only increases the energy difference between the two forms.

Target XXXIII is the only blind test target for which the ranking performance of AIMNet2 was not on par with DFT, although it still performed considerably better than the MLIPs used by other participating groups. The poor performance of AIMNet2 for this target compared to Target XXVII, Target XXXI, and Target XXXII could be attributed to the different training procedure, which did not include crystal structure generation for *n*-mer extraction and active learning. Additionally, accurately capturing long-range Coulomb interactions in salts remains a difficult challenge, representing a potential area for improvement in the AIMNet2 training. In the future, we intend to improve the training procedure for salts and cocrystals by incorporating a more sophisticated treatment of charged and long-range interactions. Overall, system-specific AIMNet2 potentials provided accuracy on par with DFT at a significantly lower computational cost. The results of the seventh CCDC CSP blind test position system-specific AIMNet2 potentials as an attractive alternative to DFT for CSP workflows.

## Conclusion

In summary, in the seventh CCDC CSP blind test we have successfully demonstrated a CSP workflow based on target-specific second generation atoms-in-molecules neural network potentials, AIMNet2. System-specific AIMNet2 potentials were trained on data sets of gas phase dispersion-inclusive DFT data acquired for molecular clusters (*n*-mers). The training sets of *n*-mers were carefully curated using active learning. These system-specific potentials trained on *n*-mers demonstrated good transferability to crystals, thus avoiding the need for expensive periodic DFT calculations for training data acquisition.

In the structure generation phase of the blind test, the computational efficiency of AIMNet2 enabled conducting geometry optimization and energy ranking of millions of structures generated by Genarris. Our team achieved the highest success rate of academic groups and third overall with 4 out of 6 possible structures generated for the targets we submitted (67%). The structures we did not generate were the solvent-stabilized Form C of Target XXXI, which was not generated by any of the blind test participants, and the structure of Target XXIX (not discussed here), which has three molecules in the asymmetric unit (*Z*′ = 3), and was only generated by one group. In the future, our CSP workflow could be improved by automating and streamlining the training of system-specific AIMNet2 potentials, and by addressing the overprediction problem with clustering.

In the ranking phase of the blind test, system-specific AIMNet2 potentials demonstrated performance that was overall comparable with dispersion-inclusive DFT methods for both geometry optimization and energy ranking. It is noteworthy that the high computational efficiency of AIMNet2 made it possible to perform fast free energy evaluations accounting for vibrational contributions and thermal expansion within the quasi-harmonic approximation for thousands of crystal structures with large unit cells. The performance of AIMNet2 was superior to the MLIPs used by other groups, which showed lower ranking accuracy and occasionally encountered problems during geometry relaxations (we are unable to analyze the reasons for the poorer performance of these models, as they are not publicly available). In the future, the performance of AIMNet2 could be improved for salts and cocrystals by developing a tailored training procedure.

Finally, based on the results presented here and in the blind test papers,^36,37^ we conclude that system-specific AIMNet2 machine-learned interatomic potentials have tremendous prospects as an efficient and accurate alternative to dispersion-inclusive DFT in CSP workflows.

## Supplementary Material



## Notes

The Supporting Information includes a description of the computational details for the DFT method, convergence information for crystal geometry optimizations, molecular volume parity plots prepared using AIMNet2 vs DFT optimized structures, and unit cell volume expansion plots for the experimental forms of different targets. ML models and submission structures for both phases (structure generation and structure ranking) of the seventh CCDC CSP blind test are available at https://github.com/isayevlab/csp7-blindtest-group16-MaromIsayev. The training data for the ML models in both phases can be found at https://figshare.com/s/bba111ee4289adc08201. For more Supporting Information on the seventh CCDC CSP blind test, please refer to the Supporting Information (SI) accompanying the main blind test publications. The Phase I and II reports include four (A–D) and three (A–C) SI sections, respectively, containing additional tables and figures of the generated structures, methodological details and group-specific analyses, experimental data, as well as relevant structural files (PDF)
