# Supplementary material for: Efficient Molecular Crystal Structure Prediction and Stability Assessment with AIMNet2 Neural Network Potentials
Source: Cryst Growth Des. 2025 Oct 14;25(21):9092–106. doi: 10.1021/acs.cgd.5c01001 (PMC12593401; doi:10.1021/acs.cgd.5c01001)
Supplement: Supplementary file 1 [file cg5c01001_si_001.pdf]

Supporting Information

Efficient Molecular Crystal Structure Prediction and Stability Assessment with AIMNet2 Neural Network Potentials

Kamal Singh Nayal,[†] Dana O'Connor,[‡] Roman Zubatyuk,[†] Dylan M. Anstine,[†] Yi Yang,[‡] Rithwik Tom,[¶] Wenda Deng,[‡] Kehan Tang,[‡] Noa Marom,[*,†,‡,¶] and Olexandr Isayev[*,†,‡]

†*Department of Chemistry, Carnegie Mellon University, 5000 Forbes Ave, Pittsburgh, PA, United States*

‡*Department of Materials Science and Engineering, Carnegie Mellon University, 5000 Forbes Ave, Pittsburgh, PA, United States*

¶*Department of Physics, Carnegie Mellon University, 5000 Forbes Ave, Pittsburgh, PA, United States*

E-mail: nmarom@andrew.cmu.edu; olexandr@olexandrisayev.com

Contents

Computational Details **S-3**

Results **S-3**

Computational Details

Convergence Criteria

The convergence of the fixed-volume variable-cell geometry optimization was determined based on the following criteria: (1) an interaction energy change of less than 4.2×10^{-3} kJ/mol, (2) root-mean-squared (RMS) and maximum atomic forces below 10^{-3} and 5×10^{-4} eV/Å, respectively, (3) RMS and maximum coordinate displacements under 5×10^{-4} and 10^{-3} Å, respectively, and (4) a maximum change in the crystal structure cell of less than 10^{-3} Å.

Results

Table S1: Overview of the training details for target-specific AIMNet2 models and the final count of optimized crystal structures for each target. The values under the Training Set column represent the sum of monomers, dimers, trimers, tetramers, and octamers in the final composition (only those present for each target).

**Target**	**AL Iterations**	**Training Set** ($\times 10^4$)	**Optimized CS**($\times 10^6$)
XXVII	8	9.50	5.0 (Phase I)
XXXI	4	22.13	3.5 (Phase I)
XXXII	4	0.62	N/A (Phase II)
XXXIII	0	2.25	N/A (Phase II)

Table S2: Overview of the crystal structure search during the structure generation phase using Genarris. The table presents the attempted targets, Z searched for each target, the total number of structures generated, and whether the experimental form(s) were identified. It can be observed that all but one experimental form, the Form C for Target XXXI, were generated by Genarris.

**Target**	Z **space**	**Total Structures Generated** ($\times 10^6$)	**Expt. Identified (Y/N)**
XXVII	{2, 4}	5.0	Y
XXXI	{2, 4, 8}	1.7	Y(A_{maj}, A_{min}, B), N(C)

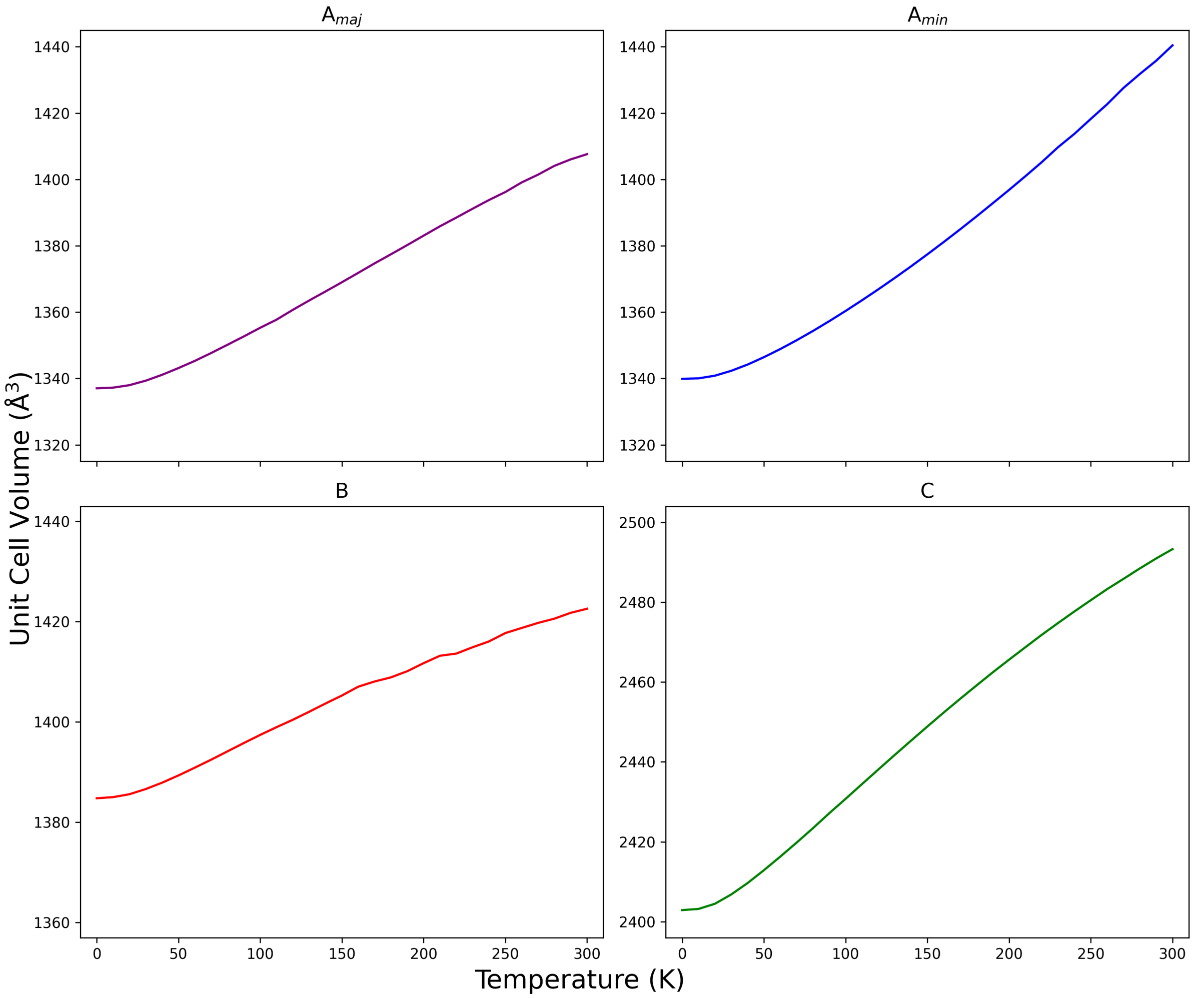

Figure S1: Unit cell volume expansion from V_{el} to V_{RT} for the experimental forms of Target XXXI.

Figure S2 presents the free energy analysis within QHA for Targets XXXI and XXXII, performed using AIMNet2 on a set of 16 distinct volume-constrained structures at 300 K, which was used to determine the Helmholtz free energy (F) as a function of volume (V). Depending on whether 100 or 500 structures were provided in the second phase, 1600 or 8000 Phonopy calculations were performed per target. Thermal corrections and other temperature-dependent properties were subsequently obtained. AIMNet2 shows reasonable agreement with DFT across the volume range, though minor irregularities are observed in the free energy curves—likely due to the complex crystal potential energy surface causing

non-uniform scaling under unit cell distortions. Incorporating more diverse, non-equilibrium molecular cluster (n-mer) configurations into the training process for target-specific AIMNet2 MLIPs may help address this non-smooth behavior.

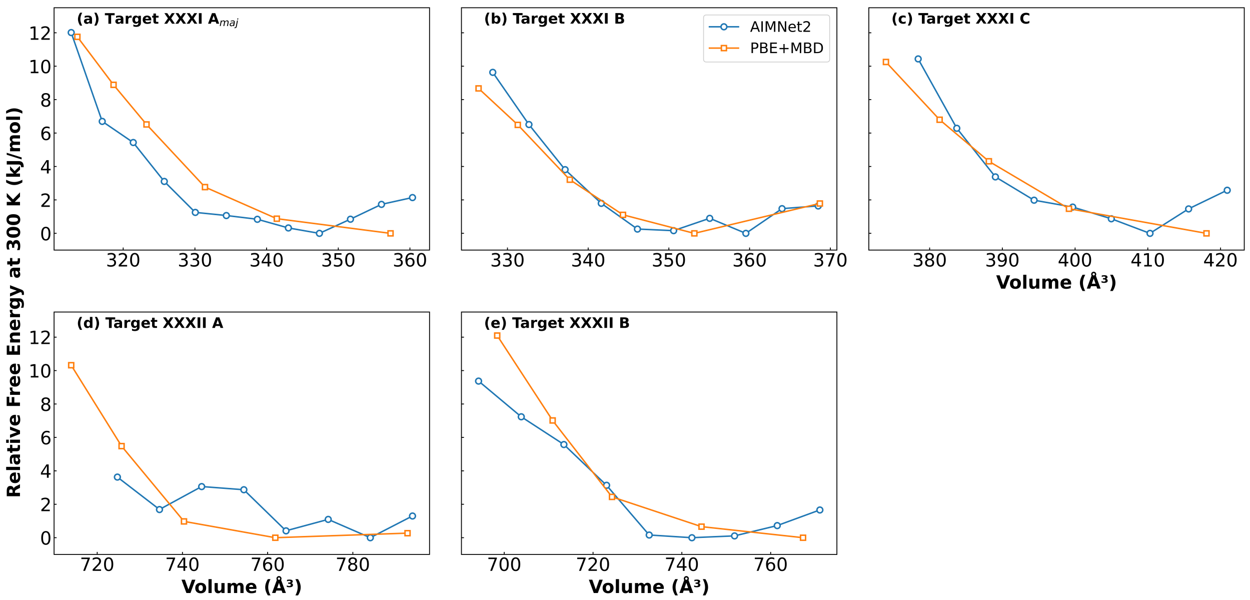

Figure S2: Relative free energy analysis for (a) Target XXXI Form A$_{maj}$, (b) Target XXXI Form B (c) Target XXXI Form C, (d) Target XXXII Form A, and (e) Target XXXII Form B.

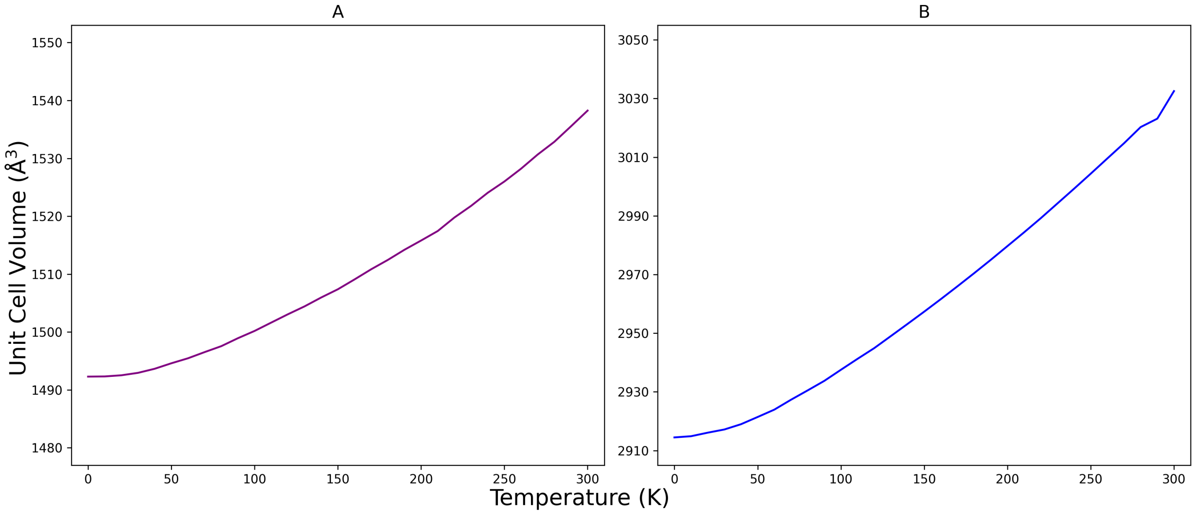

Figure S3: Unit cell volume expansion from V_{el} to V_{RT} for the experimental forms of Target XXXII.

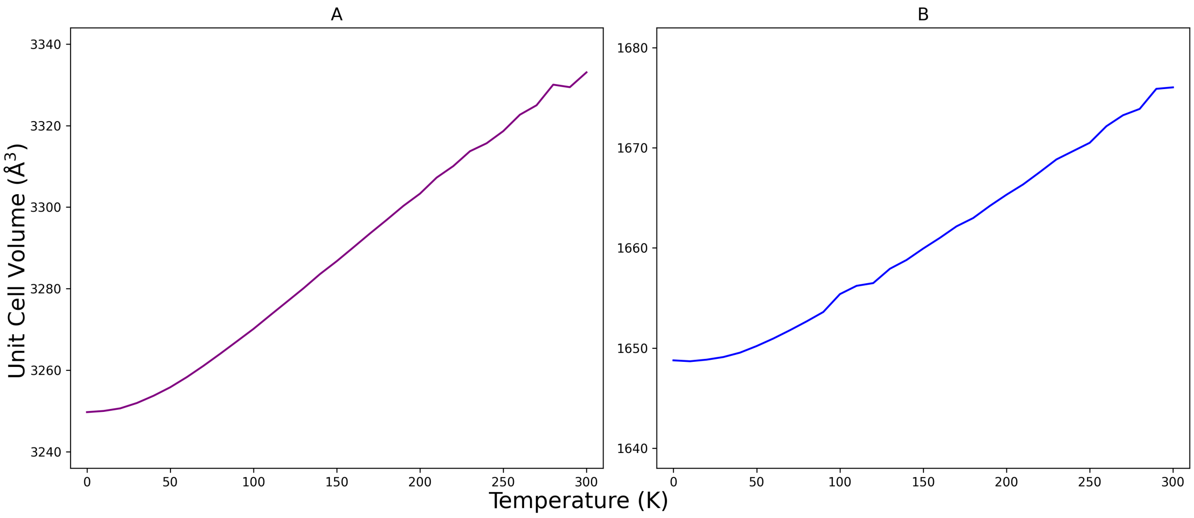

Figure S4: Unit cell volume expansion from V_{el} to V_{RT} for the experimental forms of Target XXXIII.
